# The Multifunctions and Future Prospects of Endophytes and Their Metabolites in Plant Disease Management

**DOI:** 10.3390/microorganisms10051072

**Published:** 2022-05-23

**Authors:** Yandong Xia, Junang Liu, Cang Chen, Xiuli Mo, Qian Tan, Yuan He, Zhikai Wang, Jia Yin, Guoying Zhou

**Affiliations:** 1 Key Laboratory of National Forestry and Grassland Administration on Control of Artificial Forest Diseases and Pests in South China, Hunan Provincial Key Laboratory for Control of Forest Diseases and Pests, Key Laboratory for Non-Wood Forest Cultivation and Conservation of Ministry of Education, College of Life Science and Technology, Central South University of Forestry and Technology, Changsha 410004, China; xiaxyd@126.com (Y.X.); kjc9620@163.com (J.L.); 18776202707@163.com (X.M.); nian52199@163.com (Q.T.); jassyyouz@163.com (Y.H.); wwzk621@163.com (Z.W.); 2 College of Life Science, Hunan Normal University, Changsha 410081, China; chencang2023@163.com

**Keywords:** endophytes, metabolites, biological control, multi-omics, plant diseases

## Abstract

Endophytes represent a ubiquitous and magical world in plants. Almost all plant species studied by different researchers have been found to harbor one or more endophytes, which protect host plants from pathogen invasion and from adverse environmental conditions. They produce various metabolites that can directly inhibit the growth of pathogens and even promote the growth and development of the host plants. In this review, we focus on the biological control of plant diseases, aiming to elucidate the contribution and key roles of endophytes and their metabolites in this field with the latest research information. Metabolites synthesized by endophytes are part of plant disease management, and the application of endophyte metabolites to induce plant resistance is very promising. Furthermore, multi-omics should be more fully utilized in plant–microbe research, especially in mining novel bioactive metabolites. We believe that the utilization of endophytes and their metabolites for plant disease management is a meaningful and promising research direction that can lead to new breakthroughs in the development of more effective and ecosystem-friendly insecticides and fungicides in modern agriculture.

## 1. Introduction

Plant diseases, caused by agricultural pests and pathogens, commonly result in crop losses and are a significant threat to food security. Agrochemicals are efficient in plant disease management. However, the intensive application of chemical fertilizers and pesticides has negative effects on the ecosystem and human beings, causing environmental pollution, pathogen resistance, and ecological imbalance [1]. Biocontrols, unlike chemical methods, are environmentally friendly through the action of natural control agents, such as beneficial microorganisms and their products, metabolites [2]. Endophytes and their bioactive metabolites have received considerable attention due to their potential as biological control agents (BCAs) [3,4]. 

Endophytes, including endophytic fungi, endophytic bacteria, and endophytic actinomycetes, exist in various organs, tissues, and intercellular spaces of plants without causing immediate signs of diseases [5,6]. They have established a mutually beneficial relationship with host plants during long-term coevolution. Plants provide nutrients for endophytes, while endophytes contribute to maintaining the health of plants [7,8]. Compared with soil microorganisms, endophytes within plants may have more positive and direct impacts on plants because of their special ecological niches. The results from different research teams have provided good evidence for the effects of endophytes on plant growth promotion, stress mitigation, and disease resistance in host plants [9,10,11,12]. The mechanisms proposed for disease prevention include competition with pathogens for niche and nutrition, induction of plant resistance, secretion of bioactive metabolites, and promotion of plant growth, usually working in concert [13,14,15]. Endophytic microorganisms, such as *Bacillus*, *Burkholderia*, *Enterobacter*, *Pseudomonas*, *Streptomyces*, etc., are used as microbial formulations against various phytopathogens [16].

Endophytes can produce metabolites with a variety of biological activities, such as alkaloids, polypeptides, polyketides, terpenoids, etc., which are of great significance and value in different fields, especially in agriculture and the pharmaceutical industry [14,17,18]. These metabolites have attracted a lot of attention and increased interest, because they can serve as antibiotics, insecticidal agents, natural antioxidants, antitumor agents, antidiabetic products, and so on [19,20]. In plant health protection, the major function of these bioactive metabolites is to directly or indirectly help the host plants resist biotic and abiotic stresses. For example, some antimicrobial compounds produced by endophytes are well known for strongly inhibiting pathogens, hydrolases secreted by endophytic bacteria can decompose the cell wall of pathogens, and phytohormones released by endophytes play a vital role in plant development and stress response [21,22]. Certain microorganisms called plant growth-promoting microbes (PGPM) are well-known elicitors of prime systemic resistance in plants, and they perform this function through producing metabolites such as antimicrobials and volatile organic compounds [23]. It is worth noting that studies related to the application of metabolites from endophytes in eliciting plant resistance are still limited and need to be explored and enriched in future research. 

Overall, a precise understanding of these “biocontrol agents” is important for helping plants to overcome different stress conditions. Here, we summarize several critical mechanisms employed by endophytes, particularly their metabolites, that enhance host plant disease resistance. Using endophytes and their metabolites as biological control agents can be one of the most effective ways to improve crop quality, increase yields, and achieve sustainable agriculture under the premise of minimizing the harm to the environment and humans. 

## 2. The Concept and Types of Endophytes

The definition of an endophyte was originally proposed by De Bary (1866) as “any organism that grows within plant tissues”, which is distinguished from an epiphyte living on the surface of plants [24]. One hundred years later, Carroll (1986) proposed a new definition for endophytes as organisms that inhabit the aerial parts and living tissues of plants without causing visible infection or diseases, emphasizing the mutualistic relationship between endophytes and plants; pathogenic and mycorrhizal fungi were excluded [25]. Petrini (1991) further expanded Carroll’s definition as all organisms that colonize within plant tissues for some part of their lifecycle and do not cause symptomatic infections to the host plants, from which latent pathogens are also known as endophytes [26]. Furthermore, endophytes have been defined in several different ways [5,27]. What can be seen is that the concept of endophytes has always been controversial. The definition from Petrini is commonly used in most endophyte studies. 

The major members of endophytic fungi include *Ascomycota*, *Zygomycota*, and *Basidiomycota*. In general, endophytic fungi have been recognized as two broad groups according to the life history traits and evolutionary relatedness, namely, (1) the clavicipitaceous endophytes colonizing within some grasses and (2) the non-clavicipitaceous endophytes from asymptomatic tissues of nonvascular plants, conifers, ferns, and angiosperms [28,29]. Endophytic fungi are well known to produce various bioactive compounds for anti-inflammatory, antioxidant, anti-fibrosis and antivirus drug development. Isopestacin, extracted from the fungal endophyte *Pestalotiopsis microspore*, has pronounced antioxidant properties [30]. Taxol (paclitaxel), an effective and important anticancer drug, is extracted from *Taxomyces andreanae* [31]. Another example is Podophyllotoxin, synthesized from *Alternaria tenuissima*, which exhibits excellent antitumor activity [32].

Endophytic bacteria belong to a diverse group of species, ranging from gram-positive to gram-negative bacteria, such as *Bacillus*, *Agrobacterium*, *Brevibacterium*, *Pseudomonas*, etc. [33]. The recent study conducted by Liu et al., revealed that a wide variety of endophytes inhabited wild rice, among which *Proteobacteria*, *Bacteroidetes*, and *Firmicutes* were dominant [34]. The diversity of endophytic bacteria in plants is affected by the host and environment related factors, e.g., plant growth stages, geographical location, and climatic conditions [35]. 

Endophytic actinomycetes are commonly isolated from a variety of plants, particularly from mangrove plants and medicinal plants in tropical rain forest [36]. Generally, the number and species of endophytic actinomycetes in plant roots are more than those in other parts of plants. *Streptomyces* and *Micromonospora* are the dominant genera and have been acknowledged as valuable resources for antibiotics as well as other bioactive metabolites, such as Munumbicin D, produced by *Streptomyces* NRRL 30562, and coronamycin, derived from *Streptomyces* sp. MSU-2110 [37]. Numerous species of endophytic microbes still need to be explored and identified. 

## 3. Multifunctions of Endophytes and Their Metabolites in Plant Disease Management

**Figure 1:**
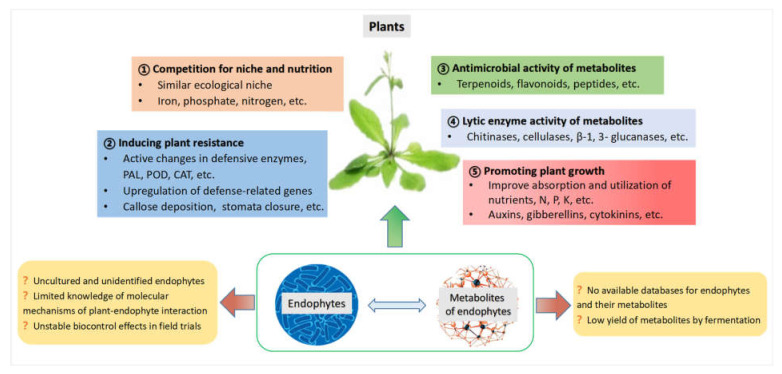
Multiple mechanisms employed by endophytes and their metabolites in plant disease management.

Endophytes secrete various metabolites that directly or indirectly enhance the tolerance of the host to different stresses, thus making them beneficial to the plants, and they potentially serve as promising biological agents in controlling plant diseases. For example, *Pyricularia oryzae* Cav., a widely studied pathogenic fungus in rice blast can be efficiently controlled by the application of endophytic microbes [38]. The fungal endophytes of *Populus alba* enhanced the host’s tolerance to the pathogen *Venturia tremulae,* as described by Martínez-Arias et al. [39]. Romeralo et al., isolated several endophytes and proved their ability to protect Aleppo pine (*Pinus halepensis*) against *Gremmeniella abietina* [40,41]. The infection and colonization of European ash (*Fraxinus excelsior*) by the pathogen *Hymenoscyphus fraxineus* could be affected by endophytic microbes via toxin secretion and/or activation of the host defense response [42]. Researchers are increasingly interested in this field, and the biocontrol roles of endophytes and their metabolites against diseases have been discussed and reported in the literature from time to time. In this section, we summarize the key mechanisms of (1) competing with pathogens for niche and nutrition, (2) producing antimicrobial compounds, (3) secreting lytic enzymes, (4) inducing systemic resistance in host plants, and (5) producing plant hormones and plant growth-promoting regulators. There are still more problems to be solved. An overview of the main functions, future prospects, and challenges in using endophytes and their metabolites in plant disease management is shown in Figure 1. 

### 3.1. Competition with Pathogens for Niche and Nutrition

Colonization in plant tissues is one of the basic properties for endophytes [43]. Endophytes generally enter the host plant in the form of thalli or spores through epidermal penetration or stomata entry, which is similar to the way pathogenic bacteria infect plants. These beneficial “micro-guests” may preferentially occupy the invasion sites of pathogens in plants and utilize nutrients, thereby reducing pathogen invasion [28]. An early case in point is that a control system using the bacterial endophyte *Bacillus subtilis* (Ehrenberg) Cohn developed by Bacon et al. [44] showed great promise for reducing invasion and mycotoxin accumulation of *Fusarium moniliforme*. The reason was that these two microorganisms occupied a similar ecological niche in maize [44]. Simultaneously, endophytes compete with pathogenic microbes for nutrition, which tends to slow the growth of pathogens. The secretion of siderophores, peptides that have high affinity for iron, is good evidence for nutrient competition. This strategy was employed by some *Pseudomonas* species in biocontrol of carnation fusarium wilt caused by *Fusarium oxysporum* f. sp. *dianthi* (Fod) [45]. That is to say, these beneficial microbes of host plants are capable of competing for nutrients and resources when they inhabit the same ecological niche as pathogens. Similarly, the study carried out by Zeng et al. [46] confirmed that the strong antagonistic activity of the rice endophyte *Streptomyces sporocinereus* OsiSh-2 towards *Magnaporthe oryzae* was associated with the competition for iron. More examples of endophytes used in biological control of plant diseases are shown in Table 1.

Competition with pathogenic microorganisms for niche and nutrition, namely niche exclusion, is a promising mechanism for the use of endophytes in plant disease control [47]. Even so, limitations could be encountered, and it might be ineffective when disease is caused by a high presence of pathogens, which was further buttressed in the study of Lahlali [48]. Strategies to solve this problem include (i) prior and extensive inoculation of endophytes to host plants through various approaches, such as seed coating, soil drench, root dip, and foliar spray application and (ii) a combination of suitable endophytes or microbes instead of individual ones [49,50].

**Table 1:** Endophytes and their metabolites used in biological control of plant diseases.

Metabolites/Compounds	Endophytic Strain	Host Plant/Isolated From	Properties/Mechanisms	References
ND	Ten endophytes functionally annotated	Pine	Niche exclusion	[51]
ND	*Bacillus cereus* BCM2, *B. cereus* SZ5, *B. altitudinis* CCM7 etc.	Strawberry, persimmon, chili, tomato	Niche exclusion	[52]
ND	*Pyrenochaeta cava*, *M. nivalis* var. *neglecta*	Elm	Niche exclusion	[53]
ND	*Burkholderia gladioli* E39CS3	*Crocus sativus* Linn.	Inducing plant resistance	[54]
ZhiNengCong, ZNC	*Paecilomyces Variotii* SJ1	Tobacco	Inducing plant resistance	[55]
ND	*Bacillus* sp. 2P2	Tomato	Inducing plant resistance	[56]
Antimicrobial compounds, cell wall degradation enzymes, etc.	*Streptomyces albidoflavus* OsiLf-2	Rice	Inducing plant resistance; lytic enzyme activity; antimicrobial activity	[57]
Hydrolytic enzymes, protease, siderophore, IAA, etc.	*Klebsiella pneumoniae* HR1	Vigna mungo L.	Inducing plant resistance; lytic enzyme activity; promoting plant growth	[58]
Antimicrobial compounds	*Pseudomonas viridiflava*	Canola	Antimicrobial activity; inducing plant resistance	[59]
Antifungal compounds	*Pseudomonas aeruginosa* H40, *Stenotrophomonas maltophila* H8, *Bacillus subtilis* H18	*P. sativum*, *B. oleracea*, *C. annuum*	Antimicrobial activity; inducing plant resistance	[60]
Antimicrobial compounds	*Penicillium*, *Colletotrichum*, *Diaporthe*, *Daldinia*, *Alternaria*, *Didymella*	*Zanthoxylum simulans* Hance	Antimicrobial activity	[61]
Eugenol, myristaldehyde, lauric acid, caprylic acid	*Neopestalotiopsis* sp., *Diaporthe* sp.	*Cinnamomum loureiroi*	Antimicrobial activity	[62]
Ethyl acetate, chloroform, methanol	*Proteus mirabilis*, *Bacillus*	*Moringa peregrina*	Antimicrobial activity	[63]
Erythromycin, ketoconazole, fluconazole, chloramphenicol etc.	*Streptomyces olivaceus* BPSAC77, *Streptomyces* sp. BPSAC121 etc.	*Rhynchotoechum ellipticum*	Antimicrobial activity	[64]
Volatile substances	*Pseudomonas putida* BP25	Black pepper	Antimicrobial activity	[65]
Antifungal compounds	*Phomopis cassia*	*Cassia spectabilis*	Antimicrobial activity	[66]
Lipases, proteases, amylases, cellulases, pectinases, xylanases	*Pseudomonas*, *Micrococcus*, *Paenibacillus*, *Streptococcus*, *Curtobacterium*, *Chryseobacterium*, *Bacillus*	Some poaceae plants	Lytic enzyme activity	[67]
Amylase, protease, cellulase, pectinase, lipase	*Doritis pulcherrima*, *Dendrobiuma phyllum*, *Dendrobium anosmum*, *Ascocentrum curvifolium*, *Aerides falcata*	Thai orchids	Lytic enzyme activity	[68]
Proteolytic enzymes, cellulase	*Phoma putaminum*, *Penicillium*, *Myrmecridium schulzeri*	*Bauhinia forficata*	Lytic enzyme activity	[69]
Chitinase	*Streptomyces* sp. P4	Sweet pea	Lytic enzyme activity	[70]
IAA	*Staphylococcus pasteuri* MBL_B3; *Kocuria* sp. MBL_B19 etc.	*Corchorus olitorius*	Promoting plant growth	[71]
Siderophore, IAA	*Ralstonia* sp.	*Poaceae*	Promoting plant growth	[72]
Siderophore, IAA, gibberellic acid	*Streptomyces* spp.	*Terfezia leonis* Tul	Promoting plant growth	[73]
Gibberellins	*Bacillus amyloliquefaciens* RWL-1	Rice seeds	Promoting plant growth	[74]
Indol acetic acid	*B. subtilis* NA-108	*Fragaria ananassa*	Promoting plant growth	[75]

ND: no data.

### 3.2. Induction of Plant Disease Resistance

Two critical patterns of plants in response to attacks of parasites or pathogens are induced systematic resistance (ISR), which is generally dependent on jasmonic acid (JA) and ethylene (ET) signaling, and systemic acquired resistance (SAR) that is commonly dependent on salicylic acid (SA) signaling. The JA/ET pathway mainly controls resistance to necrotrophic pathogens, while resistance to biotrophic pathogens is mediated by the SA pathway [76,77,78]. As described by Kloepper and Ryu, ISR mediated by some endophytes could be dependent on the SA pathway instead of the JA or ET pathways, and the signaling crosstalk between these pathways indicates that ISR is not fully separated from SAR [79]. Other plant hormones such as methyl jasmonate (MeJA) and brassinosteroids (BRs) are also involved in the plant defense system [76,80].

It has attracted a lot of interest and attention that endophytes control plant diseases through inducing plant resistance. Endophyte-inoculated plants usually have a stronger immunity to pathogens compared to uninfected plants. Utilization of endophytes in a certain part of the plant leads to a significant reduction in the disease index even though pathogens are infected in different parts of the host. The fungal endophytes *Penicillium citrinum* LWL4 and *Aspergillus terreus* LWL5 of the sunflower family (*Helianthus annuus* L.) visibly enhanced host resistance to stem rot caused by *Sclerotium rolfsii* through the SA and JA signaling networks [81]. The bacterial endophyte *Azospirillum* sp. B510, isolated from rice (*Oryza sativa* cv. Nipponbare), triggered host systemic resistance against rice blast disease and bacterial blight [82]. Similarly, the *Bacillus* strain YC7010^T^ isolated from rice by Chung et al., was developed as a novel BCA against rice bacterial blight [83]. Representative examples of endophytic strains in eliciting plant resistance are shown in Table 1.

Endophytes play an important role in plant disease control, because they can trigger plant resistance through upregulation of defense-related genes (pathogenesis-related genes such as *PR1*, *PR2*, and *PR3*, phenylpropanoid pathway genes such as chalcone synthase *CHS*, and phenylalanine ammonia-lyase gene *PAL* involved in phytoalexin biosynthesis, etc.), modifications of plant cell walls (callose deposition, stomata closure, etc.), and enhanced levels of defense-related antioxidant enzymes [84,85,86]. High levels of polyphenol oxidase (PPO), peroxidase, and phenylalanine ammonia lyase (PAL) were observed in tomato plants treated with two endophytic strains that activated systemic resistance in the host plant against Fusarium wilt [87].

Moreover, as mentioned in some reports, metabolites of endophytes can not only inhibit pathogenic microorganisms but also induce the expression of plant defense-related genes and activate the host immune response [7,88]. ZhiNengCong (ZNC), extracted from endophytic fungi *Paecilomyces Variotii* SJ1, has been proven to be an ultrahigh activity immune inducer in tobacco in a recent study [55]. More metabolites from endophytes are yet to be exploited as elicitors, and these bioactive components could provide a promising alternative resource for plant disease management. Attention has already been given to the secondary metabolites of certain non-endophytic microbes, since they have been confirmed as elicitors of plant resistance. For example, *Bacillus amyloliquefaciens* SQR9 isolated from cucumber rhizosphere produced secondary metabolites, such as fengycin, surfactin, and 2,3-butanediol, and could elicit systemic resistance in *Arabidopsis* through different signaling pathways [89]. C15 surfactin A, the major secondary metabolite of *Bacillus velezensis* HN-2 isolated from soil, not only showed strong antibacterial activity against *Xanthomonas oryzae* pv. Oryzae (Xoo) but also effectively initiated rice resistance to pathogens [90]. Another interesting example is the production of glycoprotein GP-1 obtained from *Streptomyces* sp. ZX01 isolated from soil that triggered early plant immune responses in tobacco [91]. These findings are critical for the application of metabolites from endophytes in stimulating plant resistance. 

### 3.3. Antimicrobial Properties of Metabolites from Endophytes

Endophytes are well known for their potential to produce a large number of secondary metabolites with antifungal and antibacterial properties that can directly suppress pathogens [92]. In 1993, Stierle et al., isolated an endophytic fungus from Pacific yew-Taxus brevifolia and found that it could produce the same substance as the host, thus inspiring researchers to find biologically active ingredients from plant endophytes [93]. The lipopeptide antibacterial components derived from the fermentation broth of the Chinese medicinal *Ginkgo biloba* endophytic strain *Bacillus amyloliquefaciens* CGMCC 5569 inhibited growth of *Lasiodiplodia rubropurpurea* and *L. theobromae* [94]. The results obtained by Mousa et al. [95] indicated that an endophytic fungus, WF4, isolated from the finger millet crop exhibited notable antagonist activity towards *F. graminearum* because of the production of four antifungal compounds. Endophytic actinomycetes are among the most extensively studied “producers” of antibacterial substances. Four major compounds with antibacterial activity against *Staphylococcus aureus* were obtained from the endophytic actinomycete strain LGMB491 (closely related to *Aeromicrobium ponti*) that was isolated from *Vochysia divergens*, a medicinal plant in Pantanal, Brazil [96]. Endophytic *Bacillus* and *Streptomyces* isolated from diverse environments are exploited as the most abundant antimicrobial compounds producers among Gram-positive bacteria [97]. Endophytic *Bacillus* has also been reported to produce surfactins, iturins, and fengycins [98]. The bioactive metabolites produced by endophytes are shown in Table 1.

Over the past decades, different bioactive metabolites such as terpenoids, flavonoids, peptides, and alkaloids have been exploited from endophytes for their properties to inhibit phytopathogens. Altersetin, a kind of alkaloid produced by endophyte *Alternaria* spp., displayed strong potential in inhibiting many pathogenic gram-positive bacteria [99]. Volatile substances (methanethiol, ketones, etc.) produced by *Panax notoginseng*-associated endophytic *T. gamsii* YIM PH30019 prevented the growth of pathogenic fungi [100]. The crosstalk between homologous gene clusters of endophytes and host plants could lead to production of novel metabolites; yet, the mechanisms involved still need to be identified [101]. Studies are still ongoing to explore endophytes and their metabolites for possible use in plant disease management.

### 3.4. Lytic Enzyme Activity of Metabolites from Endophytes

Endophytes are isolated from the seeds, roots, stems, leaves, or other tissues of the host plants. They produce various enzymes such as chitinases, cellulases, β-1, 3- glucanases, pectinases, glucanases, and proteases [102,103,104]. These enzymes can degrade the cell wall of pathogens or inhibit spore germination. This is an effective way to suppress phytopathogens and enable the host to obtain protection from biotic stress. In the study of Zhu and She, 45 endophytic bacteria were isolated from the *Ammodendron bifolium* plant, 40% of which showed significant activities for amylase and cellulose production, 13.3% and 53.3% of which exhibited protease and lipase activity, respectively [105]. An endophytic isolate of *Actinoplanes missouriensis* was reported to secret large amounts of chitinase, and inhibited the growth of the pathogen *Plectosporium tabacinum* by degrading the hyphae and causing plasmolysis and cell wall lysis [106]. *Streptomyces* produced several lytic enzymes that acted as antagonizing agents in *M. perniciosa* in cacao Witches’ broom disease [107]. More examples of endophytic microbes reported to have lytic enzyme production are presented in Table 1**.**

The chitinase genes of some biocontrol bacteria have been cloned and introduced into plants to improve host disease resistance [108]. Achari et al., reported that an eggplant (*Solanum melongena* L.) endophyte *Bacillus cereus* XB177R was able to produce endoglucanase and pectinase enzymes to facilitate colonization in host plants [109]. Even though many endophytes have been isolated and identified for increasing host resistance against phytopathogens because of their ability to produce metabolites with lytic enzyme activity, it remains unknown whether the lytic enzymes act as elicitors of plant systemic resistance in controlling pathogens. Mostly, these enzymes may present much stronger antagonistic activities when they are integrated with other mechanisms. Metabolites derived from endophytes are good alternative sources for numerous extracellular hydrolytic enzymes, and microbial production of enzymes is an exciting prospect for building sustainable agriculture systems [110].

### 3.5. Promotion of Plant Growth by Metabolites from Endophytes

Resistance of host plants to different stresses can be enhanced along with the promotion of plant growth and is recognized as one of the strategies employed by plants in response to pathogen attacks [111]. It is well known that endophytes and their metabolites act as promotors of plant growth. On one hand, endophytes substantially improve plant absorption and utilization of nutrients, such as nitrogen (N), phosphorus (P), and potassium (K). In particular, endophytic diazotrophic bacteria associated with gramineous crops convert atmospheric nitrogen into ammonia by nitrogen fixation; in this way, they stimulate host growth and disease resistance. Studies showed that corn seedlings inoculated with *Paenibacillus polymyxa* P2b-2R from lodgepole pine seedlings obtained 30% of foliar nitrogen from the atmosphere, and the seedling length increased by 52% [112]. On the other hand, endophytes have also been reported to accelerate plant growth by producing substances such as auxin, ethylene, gibberellin, and cytokinin. Endophytic bacteria of *Staphylococcus*, *Azotobacter,* and *Azospirillum* produce secondary metabolites, including a variety of plant hormones, which can regulate and promote the growth and development of host plants [113]. Shan et al. [114] isolated a total of 46 actinomycetes from tissue samples of 15 tea cultivars, the majority of which were able to produce IAA. More growth-promoting endophytes have been found to be common in different plants [115,116,117,118] (Table 1). Therefore, it is believed that the plant growth promotion initiated by endophytes can indirectly protect host plants against pathogens. 

In general, growth regulators and phytohormones are commonly extracted from plants or chemically synthesized in the agriculture industry. Microbial fermentation has been considered a more convenient and powerful tool to increase productivity and minimize production costs of plant metabolites. Even through numerous reports have shown successes in the production of plant metabolites by endophytes in vitro, few products have been commercially produced on a mass scale. At the same time, we must address the issue of whether bioactive metabolites are produced by the endophytes or by host plants. We lack a complete understanding of plant–endophyte interaction mechanisms. When endophytes are cultured in vitro isolated from the host, the native plant–endophyte network is correspondingly disrupted. Elaborations of the relative roles of the “host plants” and the “micro-guests” in the production of certain metabolites need to be studied in greater depth. 

## 4. Application of Endophytes and Their Metabolites as Novel BCAs in Agriculture

Endophytes that colonize within a plant without causing symptoms of diseases and also have the ability to produce abundant bioactive metabolites are potential biological control agents [119]. They are appropriate substitutes for agrochemicals to minimize the impact on the environment and to ensure a sustainable agricultural system. Many studies on the exploitation of endophytes for biological control of plant diseases have been carried out in recent years. In a previous study, using *Epichloë typhina* isolated from Timothy-grass reduced the disease incidence of host plants caused by *Cladosporium phlei*; this was the first record of fungal endophytes used for the biocontrol of foliar cereal diseases [120]. It is interesting to note that a certain endophyte has the potential to become a biological control agent (BCA), while the same species might also promote growth and improve stress tolerance of the host plants [119]. Other microorganisms such as *Bacillus* spp., *Enterobacter* spp., *Pantoea* spp., *Pseudomonas* spp., and *Streptomyces* spp. have recently been manipulated and applied for various biocontrol programs in agriculture [119,121,122]. There is still not yet a trend to exploit and commercialize endophytes and their metabolites as BACs in modern agriculture. 

The application of plant endophytes in agriculture can be achieved by microbial pesticides, that is, mass propagation of living microbes and processing into preparations for use. These novel BCAs are derived from plants, act on plants, and do not contain the toxic ingredients of traditional pesticides; so, they are environmentally friendly [123]. Many factors in the field and a plant’s micro-ecological environment can affect the performance of endophytic microbe resistance to stress and diseases. Some endophytes have been found to be highly host-specific and can only adapt to the specific tissues of specific plants [124,125,126]. Moreover, the infection rate of endophytes to non-natural host plants is also a critical issue when applied to plant disease management. Geographical factors cause physiological changes in plants, resulting in significant changes in the colonization and diversity of endophytes within the same plant in different sampling sites [127]. Therefore, the ecological, pathological, and other factors (e.g., complex life cycles of pathogens, inter-species variation/diversity/interrelationship) must be considered in the practical application of endophytic pesticides [128,129,130].

Endophytes are a rich source of diverse and functional metabolites [131]. On the one hand, natural active substances such as plant hormones, antibiotics, and alkaloids can be used to resist diseases and promote growth of plants. After separation and extraction, these compounds can be directly applied to agricultural production. On the other hand, the secondary metabolites of plant endophytes can be used as lead compounds of green pesticides (biopesticides). A recent paper noted that 51% of bioactive substances isolated from endophyte fungi were previously undiscovered, while only 38% of those isolated from soil microorganisms were [132]. Accordingly, exhaustive exploitation for endophyte metabolites with biopesticide activity is a promising direction to develop new biopesticides.

Plant endophytes in different extreme environments such as cold, drought, and high-latitude often produce secondary metabolites with special physiological functions, which are of great value to agriculture [6,133]. Therefore, specific active substances can be obtained by screening endophytes from special ecological environments. Researchers have conducted significant work on the isolation, extraction, and identification of these bioresources. At present, reports of lead compounds structures are relatively limited, and it is believed that there will be breakthrough progress in the near future.

What concerns us most is the potential toxicity of these expected products to plants, animals, and humans. In fact, both plants and animals are eukaryotes. Endophytes belong to the core microbiome in plants, as they establish a symbiotic relationship with the host. Studies have shown that most antimicrobial agents produced by endophytes are toxic to phytopathogens, eco-friendly, and do not harm plants and the human [21,134]. Plants automatically act as selection systems for nontoxic biologically active substances. Overall, the use of plant endophytes and their metabolites as sources of novel BCAs in plant disease management has obvious advantages and great potential. 

## 5. Multi-Omics Approaches for Mining Bioactive Metabolites from Endophytes

With the progress in high-throughput sequencing and systems biology, multi-omics, encompassing genomics, transcriptomics, proteomics, and metabolomics, are becoming more and more essential in plant–microbe interaction research [135,136,137]. Genomics deals with the entire genome sequences, while transcriptomics studies RNA and gene expression patterns [138,139]. Proteomics examines the structures, functions, and interactions of dynamic proteins, while metabolomics aims to understand the synthesis, decomposition, and transformation of metabolites in a particular organism [140,141,142].

Genome analysis is critically important for identification and characterization of the genes involved in the beneficial plant–endophyte interaction. It has revealed the genes responsible for their colonizing preferences within plants as well as the synthesis of various bioactive compounds, for example, genes for plant hormones and antibiotics production, nitrogen fixation, and nutrition acquisition (K, P, Fe, etc.) [143,144]. Genome analysis of *Piriformospora indica* revealed its potential as a bioactive agent for plant production [145]. Complete genome sequences of many endophytes have been summarized by Kaul et al. [146]. Multigenome analysis or genome comparison analysis has provided a new tool to unravel some of the mysteries, namely, closely related endophytic species perform different roles in host plants, and the different metabolite gene clusters illustrate the diversity of endophyte metabolites to a certain extent [147]. 

Genomics provides the genomic sequencing information, while transcriptomics links gene functions with specific conditions. The dynamics and regulation of actively transcribed genes can be achieved by transcriptomic analysis. For example, the sequencing of mRNA is a useful approach to understand the different responses of plants in the presence and absence of endophytes [148]. Proteomics is aimed at the analysis of entire proteins from a tissue or an organism under a specific condition [141]. Similarly, total protein content of endophyte-free and endophyte-inhabited plants can be extracted and assessed to investigate the specific proteins involved in the relationship between the two groups. As shown by Lery et al., 78 differentially expressed proteins related to the endophytic *Gluconacetobacter*-sugarcane interaction were identified by proteomic analysis based on mass spectrometry [149]. Yuan et al., employed transcriptomics and proteomics of the host *Atractylodes lancea* inoculated with and without endophyte *Gilmaniella* sp. AL12 to decode the effect of endophyte treatment [150].

Metabolomics, widely used in bioanalytical procedures, enables the identification and quantification of the metabolites in a sample. It can complement transcriptomic and proteomic data, thus facilitating a better understanding of host phenotypic features and unveiling the mechanisms of plant–microbe interactions [151]. Metabolomics are often classified into two groups: untargeted metabolomics, a comprehensive analysis of numerous known/unknown metabolites in the sample, and targeted metabolomics, the measurement of the specific metabolites in the sample [152]. Untargeted metabolomics can be performed to discover novel compounds, and targeted metabolomics is able to validate the results of untargeted metabolomics [153]. As described by Liu et al., the high-performance liquid chromatography-mass spectrometry (HPLC-MS) based analysis showed that 88 secondary metabolites including 70 novel natural products were produced by *Pestalotiopsis fici* isolated from healthy *Camellia sinensis* (Theaceae) [154]. Other analytical platforms for metabolomics such as liquid chromatography-mass spectrometry (LC-MS) and nuclear magnetic resonance (NMR) have been recently reviewed by Segers et al. [155].

Integrated analysis of transcriptomics and metabolomics is able to analyze both the genes and metabolites that are differentially expressed along a time curve in exploring the causal relationships between genes and metabolites. The key genes and metabolites and key signaling and metabolic pathways can be found through bioinformatic tools such as functional annotation and pathway enrichment, leading to an improved understanding of the physiological and molecular mechanisms [156,157]. In recent years, transcriptomics–metabolomics combined analysis has been widely used in studies of rice, potato, tobacco, and other plants [158,159,160,161,162]. Sade et al. [163] conducted a study on susceptible and resistant tomato plants to determining the differences in the regulated pathways and the levels of specialized metabolites between these two samples in response to tomato yellow leaf curl virus invasion using both comparative transcriptomics and metabolomics. In another example, transcriptomic and metabolomic analyses were performed by Yang et al., to detect the differentially expressed genes (DEGs) and metabolites of anthracnose-resistant and susceptible varieties of *Camellia oleifera*, suggesting the important role of flavonoid biosynthesis in the defense against anthracnose [164]. As mentioned in the report of Kaul et al. [146], differential expression analysis regarding endophyte-free and endophyte-inoculated plants can be helpful to fully understand the mechanisms of plant–endophyte interactions and endophyte-mediated disease resistance. 

**Figure 2:**
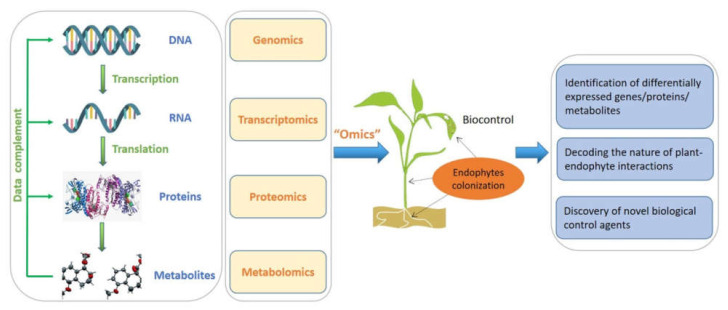
Multi-omics approaches for the research of endophytes and their metabolites in plant disease biocontrol.

In terms of plant–endophyte research, omics technology and multi-omics joint analysis will definitely continue to play irreplaceable roles not only in the study of host growth and stress tolerance but also in discovery of bioactive metabolites (Figure 2). However, multi-omics data analysis still faces great challenges. How to better correlate the data of various omics and mine more useful information remains one of the main problems that needs to be continuously explored [165].

## 6. Conclusions and Future Prospects

In the past three decades, remarkable progress has been made in research on plant disease resistance mechanisms and plant–microbe interactions. Endophytes, colonizing plant tissues, are regarded as naturally occurring agents in plant disease suppression. Most of their success is attributed to the production of a vast array of metabolites. These metabolites have an abundance of biological activities, making them a promising resource collection, and they play an increasingly important role in different fields. Multi-omics joint analysis could achieve data complementation from genes to metabolites; thus, it has been used extensively as an approach to comprehensively discover physiological and molecular mechanisms in plant disease resistance. In this review, we reported the multifunctions of endophytes and their metabolites in the biocontrol of plants. We provided extensive evidence and recent examples and described the importance of these bioresources for future agricultural development. As we have outlined, one of the mechanisms of endophytes in plant protection is the elicitation of plant resistance. In addition, various metabolites from non-endophytic microorganisms have been identified as elicitors of plant resistance. However, the current research on endophyte metabolites in biological control is mainly focused on antibacterial, hydrolase activities, and growth-promoting value, and the reports on the induction of plant resistance are relatively limited. So we propose using multi-omics approaches to isolate and identify more metabolites from endophytes, especially as plant resistance inducers, for increasing plant fitness and crop yields.

Biological control agents are reliable and environmentally friendly in plant disease management and crucial for sustainable agriculture. Using endophytes and their metabolites for plant protection has many advantages over chemical pesticides and conventional bioformulations. The metabolites of plant endophytes contain a variety of bioactive ingredients that enhance the host defense against pathogens, rather than simple toxic properties. Therefore, application of one or several natural active substances as the lead compound is among the most promising approaches for green pesticide discovery in the future. However, in order to truly achieve their large-scale commercial production and application, we still have some challenges to overcome, such as the following:

(i) Many endophytes are uncultured and unidentified.
(ii) There are no available databases for endophytes and their metabolites.
(iii) Knowledge of the molecular mechanisms of plant–endophyte interactions is limited.
(iv) Biocontrol effects of endophytes are not definitely stable in field trials.
(v) Yield of metabolites by fermentation is low.

It is necessary to elevate the exploitation of endophytes and their metabolites in the biological control of plant diseases to the multi-omics level as a promising research frontier.
